# Analysing Biases in Genealogies Using Demographic Microsimulation

**DOI:** 10.1007/s10680-025-09756-4

**Published:** 2025-12-05

**Authors:** Liliana P. Calderón-Bernal, Diego Alburez-Gutierrez, Emilio Zagheni

**Affiliations:** 1 Max Planck Institute for Demographic Research, Rostock, Germany; 2 Stockholm University, Stockholm, Sweden

**Keywords:** Genealogies, Microsimulation, Biases, Historical demography, Kinship

## Abstract

An incomplete understanding of biases affecting the representativeness of genealogies has hindered their full exploitation. We report on a series of experiments on synthetic populations assessing how structural biases in ascendant genealogies affect the accuracy of demographic estimates. Using the SOCSIM microsimulation programme and Swedish fertility and mortality data (1751–2022), we analyse three biases: lineage survival, limited coverage of collateral kin, and selective omission. Comparing demographic measures from ‘fully recorded’ and ‘bias-infused’ synthetic populations, we find that across the period, including only direct ancestors can underestimate total fertility rate (TFR) ($\approx -42\%$) and overestimate life expectancy at birth ($e_0$) ($\approx +33\%$), mainly due to missing infant, child, and some young adult deaths. Including direct ancestors’ offspring shifts TFR to overestimation ($\approx +21\%$) while improving mortality estimation across all ages, with $e_0$ overestimation reduced to $\approx +1.8\%$. Our study shows that completeness of family trees is essential for obtaining accurate demographic estimates from genealogies.

**Supplementary Information:**

The online version contains supplementary material available at 10.1007/s10680-025-09756-4.

## Introduction

Long-term analysis of demographic dynamics, especially considering generational and kinship relationships is usually challenging and data-demanding. Questions involving inter- (i.e., between) and multi- (i.e., many) generational perspectives often require data on vital events and kinship networks spanning decades or centuries. For instance, examining the familial transmission of demographic outcomes such as longevity requires long historical data series including kinship information, that allows to consider the lifetime of multiple generations. These data requirements can limit the scope of inter- and multi-generational studies to specific periods or geographic areas for which such data sources exist. However, unique opportunities for population research have recently emerged thanks to the availability of novel data sources driven by the Data Revolution (Alburez-Gutierrez et al., 2019; Kashyap, 2021), coupled with the increasing use of computationally-intensive tools such as microsimulation (Zagheni, 2015). Novel data sources, including those resulting from digitisation and crowd-sourcing of historical records, can provide opportunities to study long-term dynamics, whose analysis has often been limited by the lack of (good) historical data. Thus, comprehending the potential and constraints of available sources and appropriate methods for their use can help broaden the scope of research in historical and kinship demography.

Genealogies hold great promise for this type of analysis, as they could enable us to link human populations over time, across space and generations. However, as they often suffer from problems of coverage and representativeness (Dupaquier, 1993), a deep understanding of their characteristics, quality problems, and biases is essential for informed use in demographic studies. Missing information issues include dates of birth and death and the omission of women, children who died at an early age and people who brought dishonour to the family (Hollingsworth, 1976; Zhao, 2001). Moreover, genealogies are usually records of surviving patrilineal lineages, which generally experience better demographic conditions and show higher sex ratios than the entire population. Hence, extinct and matrilineal lineages are generally omitted from them (Zhao, 2006). Besides demographic selectivity, which can lead to underestimating mortality and overestimating fertility, individuals with high socioeconomic status are more likely to be included in genealogies (Campbell & Lee, 2002).

Large online genealogical databases have recently become more available through the collaborative efforts of users of genealogical sites, such as Family Search, Geni and WikiTree (Charpentier & Gallic, 2020).These sources have been utilised in the analysis of historical demographic patterns, with a particular focus on mortality (Gavrilova & Gavrilov, 2007; Kaplanis et al., 2018; Minardi et al., 2024), morbidity (Rawlik et al., 2019), fertility (Hsu et al., 2021; Blanc, 2022), family structures (Corti et al., 2024; Skopek et al., 2024) and migration (Blanc, 2024).

Some of these studies have also made significant methodological contributions to data quality assessment. Kaplanis et al. (2018) collected, cleaned and validated a large-scale crowdsourced genealogical dataset, FamiLinx, which has been used in subsequent studies. Stelter and Alburez-Gutierrez (2022) suggested that mortality measures from these data are non-representative of the general population and are biased towards the elite. Chong et al. (2022) proposed a Bayesian model combining structured mortality estimation and smoothing techniques to correct mortality rates. Minardi et al. (2024) validated the data against historical sources, discussed potential biases, and assessed the impact of record precision and completeness on mortality estimates over time. Blanc (2024) proposed an adjustment method for fertility estimates by selecting genealogies that are not restricted to direct lineages. Corti et al. (2024) compared the data with census records, focusing on migrant assortative mating. Colasurdo and Omenti (2024) measured the completeness and quality of demographic information at the individual and family level.

Whilst prior studies have examined certain issues in offline and online genealogies, further analysis is necessary to ascertain the accuracy of the measures derived from these data. This research contributes to this effort by offering a simulation-based approach to assessing the effect of structural biases arising from the genealogical reconstruction process on demographic measures. Although this study does not focus on any particular dataset, its findings aim to provide researchers working with genealogies with insights into how structural biases can distort demographic estimates and highlight the importance of considering the completeness of family trees.

Genealogies are traditionally divided into ascendant and descendant (Bideau & Poulain, 1984; Jette & Charbonneau, 1984; Oeppen, 1999). In both cases, the starting point is an individual so-called ‘ego’, but while the former traces their ancestors backwards in time, the latter records their family trees prospectively. Both types of genealogies may or may not include lateral kin i.e., those relatives who share a common ancestor but are not in a direct line. For historical demography, descendant genealogies have often been considered more appropriate, as they can be used directly with the family reconstitution method (Bideau & Poulain, 1984; Jette & Charbonneau, 1984; Dupaquier, 1993), and are potentially unbiased when they are complete (Oeppen, 2021). However, they are often limited in number and size and restricted to specific areas with available parish records, such as the ones used by Henry (1956); Hollingsworth (1964, 1977), population registers in Belgium or Sweden, or the genealogical records for China. Here, we focus on ascendant genealogies which, despite their structural biases, are more likely to be found outside the limited number of countries with high-quality records including kinship ties. This type of genealogy has also become more accessible through online genealogical databases.

Demographic microsimulation has proven useful for investigating long-term kinship patterns (Murphy, 2011) as well as for evaluating historical data and assessing the reliability and bias of genealogies (Oeppen, 1999; Zhao, 1994, 2001, 2006) and family reconstitutions (Ruggles, 1992). Despite some constraints of microsimulation models, such as limitations when considering demographic similarities within the same kin group (Ruggles, 1993), or the dependence of the demographic events (and their timing) on the assumptions and input parameters (Zhao, 2006), they remain a powerful tool for analysing the effects of selection and under-representation issues in genealogies. For instance, Zhao (2001) used microsimulation to compare the demographic conditions of the members of simulated male surviving patrilineages — considered to be very similar to the male lineages recorded in Chinese genealogies — with those of the members of all simulated lineages, taken as a representative sample of the entire population. The results showed that, for the first four or five simulated generations, male members of the surviving lineages had a higher average age at death, a higher proportion married, a higher average number of children and a higher proportion with sons, than males of all simulated lineages. This provided insights into the effects of selectivity in descent-type genealogies such as the Chinese, especially linked to their patrilineal nature and survival bias.

As the possibility to infer demographic dynamics from genealogical data depends on the nature and representativeness of the data, it is essential to assess the size and effect of their inherent biases before drawing conclusions about the general population. In this research, we examine biases arising from the genealogical reconstruction process, particularly in ascendant genealogies, without focusing on any specific database. Therefore, our focus is on inherent or structural biases, i.e. distortions arising from the completeness of family trees reconstructed from the present.

Although our study and Zhao (2001)’s work both use microsimulation to examine biases in genealogical data, our study extends his framework in three important ways. While Zhao assessed the impact of survival bias on demographic estimates derived from descending patrilineages, our study focuses on estimates based on ascendant genealogies, which are becoming increasingly available. Our analysis of lineage survival is also broader, incorporating both patrilineal and matrilineal lines and examining additional sources of bias related to the completeness of reconstructed family trees. Finally, whereas Zhao modelled populations using fixed demographic parameters to approximate historical conditions, we simulate demographic change over a longer period (1751–2022) using year-specific demographic rates. However, we do not account for further biases identified by the literature, such as the ‘accuracy bias’ resulting from the level of detail in the recorded information (Minardi et al., 2024) or the ‘elite bias’ arising from the oversampling of the elites who are more visible in the historical records (Stelter & Alburez-Gutierrez, 2022).

We conducted a series of experiments on synthetic populations, simulated using the SOCSIM demographic microsimulation programme (Hammel et al., 1976), and taking Sweden as a study case, which allowed us to cover several generations thanks to the availability of long-established vital statistics. Based on synthetic populations with ‘fully-recorded’ information, we replicated the construction of ascending family trees for a group of hypothetical genealogists to evaluate the effect of some typical sources of bias in such trees on demographic measures. More specifically, we aim to understand *how these sources of bias affect the accuracy of fertility and mortality estimates derived from ascendant genealogies*. Our analysis seeks to contribute to a better understanding of the possibilities and limitations of using genealogies for demographic research.

## Data and Methods

### Demographic Microsimulation

We ran demographic microsimulations over four centuries using the SOCSIM microsimulation programme and Swedish data (1751–2022) to obtain ‘fully-recorded’ synthetic populations, i.e., the register of every single individual who was ever alive during the simulation, including the information on their vital events and kinship relationships. SOCSIM is an open-source demographic microsimulation programme, originally developed at the University of California Berkeley (Hammel et al., 1976), and written in C programming language. It has been used for decades in demographic research to address issues such as kin availability and kin loss (Murphy, 2004, 2011; Verdery & Margolis, 2017; Zagheni, 2011), among others.

The microsimulator requires two inputs: an initial population file containing information on the sex and date of birth of each individual, and monthly age-specific fertility rates and age-specific probabilities of death for individuals of a particular sex, group and marital status (married, single, divorced or widowed) over a given period. Fertility rates can be parity-specific, but this is not the case in this study, since parity-specific rates are only available from 1970 onwards. However, the parity distribution generated by the microsimulation, despite not using parity-specific data, is acceptable overall, although it does not perfectly mirror a distribution derived from empirical, individual-level data. This assertion is supported by Calderón-Bernal et al. (2025), who assessed the accuracy of mean numbers and distributions of kin, derived from a microsimulation setup similar to that used in the present study, using Swedish population registers as a benchmark. During the simulation, SOCSIM schedules and executes vital demographic events (births, marriages and deaths) for each ‘living’ simulated individual in the initial population and their descendants.

A brief description of how the microsimulator works is given in Mason (2016) and summarised below. At the beginning of each simulation segment (i.e., when the demographic rates or societal constants change) or month, SOCSIM schedules an event for each living individual to be executed at a future date. Only one event can be scheduled for each individual at any one time. After a person’s event has been executed (except in the case of a death) or a change in their marital status or parity, a new event is scheduled for that person. To determine the next event to be scheduled for each individual, SOCSIM generates a random waiting time for each event for which each individual is at risk, considering the sex, age, group, and marital status-specific rates. Once all potential events have randomly generated waiting times, the event with the shortest waiting time is selected and scheduled. The event competition thus follows a competing risks framework, where the probability of experiencing each event for which the individual of a given sex, age and marital status is at risk is independent of all others. All events scheduled for a given month are executed in random order. SOCSIM then increments the month and repeats the event execution. At the end of the simulation, SOCSIM writes an output population file containing information about each individual who has ever lived, and a marriage file containing information about each marriage generated during the simulation.

We ran simulations using the ‘rsocsim’ R-package (Theile et al., 2023) and input rates from the Human Fertility Collection (HFC) (1751–1890), the Human Fertility Database (HFD) (1891–2022) and the Human Mortality Database (HMD) (1751–2022). The last two were retrieved via the ‘HMDHFDplus’ R-package (Riffe, 2015). To minimise the effects of microsimulation stochasticity without significantly compromising computational time, we ran ten simulations with the same initial population and input rates but different randomly generated seeds. This allowed us to perform the experiments, explained in the next subsection, on more than one synthetic population and then average the results. As done in previous studies using SOCSIM, we first ran the simulator for 100 years using the age-specific rates for 1751 to produce a stable age structure. This resulted in populations of about 15,000 individuals in 1751, which were then subjected to the corresponding annual rates for 1751–2022. We ran the simulator for another 50 years using 2022 rates to avoid inconsistencies in the final years, particularly mismatches between births and mid-year population counts when introducing biases. However, the experiments were conducted based on the synthetic populations alive at the end of 2022 (approximately 100,000 individuals per simulation). Due to the lack of accurate age-specific marriage rates by sex for the entire period, we used the ‘marriage after childbirth’ option in ‘rsocsim’ to simulate lifelong partnership, selecting a living single man each time a previously single woman gave birth. Despite its name, this option corresponds to lifelong partnerships, rather than real marriages. Following Alburez-Gutierrez et al. (2021), partners for each woman were selected from all living single men to minimise the squared difference between the observed distribution of ‘man’s age - woman’s age’ and a normal distribution with a mean of two and a standard deviation of three.

To assess the accuracy of our microsimulations, we estimated period age-specific fertility rates (ASFR) for women and age-specific mortality rates (ASMR) for both sexes, and their corresponding summary measures, total fertility rate (TFR) and life expectancy at birth ($e_0$), based on the 10 SOCSIM outputs to verify that they are close to the input rates and derived measures. Figure 5 in Appendix compares the estimates of ASFR, ASMR, TFR and $e_0$ derived from the simulation inputs (i.e., HFC/HFD and HMD) and outputs. As expected in a stochastic process, there is still some variation around the reference value (input rates), especially when fertility rates are higher (distant periods) and mortality rates are lower (recent periods, for infant and child mortality). This can be explained by the fact that the initial populations are smaller than the final populations after the simulated populations have grown. Nevertheless, the gaps were reduced when the summary measures (TFR and $e_0$) and the average of the ten simulations for each measure were calculated. We chose to run ten simulations, as that represents an appropriate compromise between the computational time needed to run simulations and the level of stochasticity that remains after averaging across simulations.

### Experiments on Synthetic Populations

We conducted a series of experiments on synthetic populations to assess the effects of key sources of bias in ascendant genealogies on demographic measures. Ascendant genealogies reconstruct family trees backwards in time and necessarily rely on lineage survival, meaning that only ancestors with descendants who survive to the time of genealogical reconstruction are observable. This introduces a fundamental bias into the genealogical data on which this research methodology is based. Throughout the experiments, we examined key biases resulting from the retrospective reconstruction and the varying completeness of family trees reconstructed by genealogists. Based on prior research, we identified three main sources of bias for investigation: (1) *lineage survival*, (2) *limited coverage of collateral kin*, and (3) *selective omission*. While these sources of bias can affect both ascendant and descendant genealogies, our analysis focuses on their implications for ascendant genealogies.

The first source of bias arises from the fact that genealogical reconstructions that start with individuals who are currently alive necessarily omit extinct lineages. By definition, ancestors without living descendants, such as childless individuals, are excluded. This selection process makes genealogical reconstruction dependent on *lineage survival*. The second source of bias arises from the *limited coverage of collateral kin* as genealogists often have limited knowledge of their extended family and may omit collateral kin, such as siblings, aunts, uncles, etc. This may be due to restricted information, a lack of data sources, or intentional exclusion. The third source of bias is the *selective omission* of individuals from surviving lineages, such as children who died young or childless women, due to memory loss or a lack of information. Although this type of omission does not account for all the reasons why children who died early and childless women may be missing from genealogies, we focus on the potential effects of under-reporting them within otherwise complete surviving lineages.

To evaluate these sources of bias, we replicated the genealogical reconstruction process for a sample of individuals who were alive at the end of each simulation. We considered these individuals to be our hypothetical genealogists. Specifically, we randomly selected 10% of individuals who were alive at the end of 2022 in each simulation. These individuals, hereafter referred to as ‘genealogists’, served as the starting point for reconstructing individual family trees. We then combined the complete family trees of all the genealogists in each simulation to form the ‘genealogical subsets’ used in each experiment.

We assessed the size and effect of the biases by comparing commonly used demographic measures, derived from the ‘bias-infused’ genealogical subsets with those obtained from the ‘fully-recorded’ synthetic population, which served as the benchmark. As measures of period fertility and mortality, we included ASFR, ASMR, TFR and $e_0$. We adopted the same approach across the three experiments described below (see Table 1 for a summary of the experiments). To minimise the effects of microsimulation stochasticity, we ran all the experiments across the ten simulations. We then calculated the demographic measures from both the ‘bias-infused’ and the ‘fully-recorded’ populations in each simulation, and averaged the results. As a summary measure of the bias, we calculated the absolute difference between each genealogical subset and the whole simulation, averaging the results to obtain absolute and relative mean differences.

In the first experiment, we examined genealogical subsets comprising *incomplete surviving lineages*, thereby capturing the combined effects of lineage survival and limited coverage of collateral kin (biases 1 and 2). We traced only the direct ancestors (i.e., individuals connected by parent–child relationships) of each genealogist across nine generations (from parents to 7x-great-grandparents). As multiple genealogists may share a common ancestor, combining the genealogies can introduce duplicates. This replicates a common issue in real-world genealogical data, particularly on collaborative platforms that compile the family trees from multiple genealogists. To assess the effects of both demographic selectivity and duplicate-induced inflation, we compared demographic estimates derived from this subset (both with and without duplicates) with estimates from the whole simulation.

In the second experiment, we expanded the genealogical subset to include *complete surviving lineages*, encompassing up to 1,022 direct ancestors and their offspring for each genealogist. Specifically, we included the offspring of the genealogists’ parents (i.e., siblings) and all the direct ancestors up to the level of 7x-great-aunts and 7x-great-uncles (see Table 1). We gradually added one type of kin to the genealogical subset from each simulation. However, for the sake of readability, the main text only presents the results comprising all direct ancestors and their offspring. After removing duplicates, we compared demographic measures derived from this subset of complete surviving lineages with those from the whole simulation and the incomplete surviving lineages (Experiment 1).

In the third experiment, we introduced *selective omission* into the complete surviving lineages described in Experiment 2. This experiment is divided into two parts. In Experiment 3A, we randomly removed a proportion (25%, 50%, 75%, or 100%) of children who died before the age of five over the entire simulation period (1751–2022), thereby simulating memory-related omissions of early-deceased children from complete surviving lineages. In Experiment 3B, we randomly removed a proportion (25%, 50%, 75%, or 100%) of women who reached the age of 15 but remained childless, thereby simulating the selective forgetting or exclusion of women who did not leave descendants from the complete surviving lineages. These removals resulted in *surviving lineages with selective omission*, in which the genealogical branches remains almost complete but certain individuals are excluded. After removing duplicates, we computed demographic measures from these surviving lineages with selective omission and compared them with estimates from both the complete surviving lineages (Experiment 2) and the whole simulation.

**Table 1:** Experiments to assess the effect of key sources of bias in ascendant genealogies: genealogical subsets and kin types included

Experiment	1. Incomplete Surviving Lineages	2. Complete Surviving Lineages	3. Surviving Lineages with selective omission
Source of bias	Lineage survival and limited coverage of collateral kin	Lineage survival	Lineage survival and selective omission
Effect on genealogies	Exclusion of extinct lineages and collateral kin	Exclusion of extinct lineages	Exclusion of extinct lineages and certain individuals
Genealogists	10% sample of individuals aged 18+ alive by 31.12.2022		
Genealogical subsets			
Direct ancestors	All	All	All
Direct ancestors’ offspring (collateral kin)	No	All	All
Omission of children who died before age 5	No	No	25%, 50%, 75%, 100% removed
Omission of childless women	No	No	25%, 50%, 75%, 100% removed
Kin types included			
Genealogist (ego)	All	All	All
Parents	All	All	All
Grandparents	All	All	All
1x-Great-grandparents	All	All	All
2x-Great-grandparents	All	All	All
3x-Great-grandparents	All	All	All
4x-Great-grandparents	All	All	All
5x-Great-grandparents	All	All	All
6x-Great-grandparents	All	All	All
7x-Great-grandparents	All	All	All
Siblings	No	All	All
Aunts/uncles	No	All	All
1x-Great-aunts/uncles	No	All	All
2x-Great-aunts/uncles	No	All	All
3x-Great-aunts/uncles	No	All	All
4x-Great-aunts/uncles	No	All	All
5x-Great-aunts/uncles	No	All	All
6x-Great-aunts/uncles	No	All	All
7x-Great-aunts/uncles	No	All	All

## Results

Through the series of experiments described above, we evaluated the effect of three sources of bias in ascendant genealogies on measures of period fertility and mortality. All the experiments were run independently for each of the ten simulations. The demographic measures were then calculated for each subset from each simulation, and the results were averaged. For the sake of readability, we present here the mean demographic measures derived from the whole simulated population and the genealogical subsets created for the experiments.

### Experiment 1. Incomplete Surviving Lineages

**Fig. 1:**
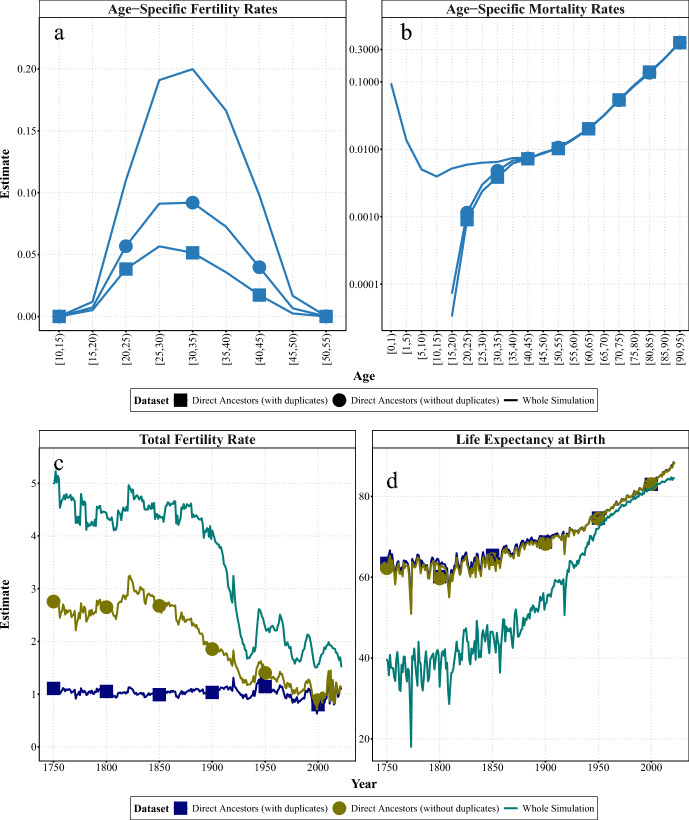
Experiment 1: Age-specific (1900–1905) and summary demographic measures (1751–2022) for women in Sweden, estimated from SOCSIM microsimulations and synthetic genealogical subsets comprising only direct ancestors (with and without duplicates).* Notes* In each panel, the figure represents the means for each dataset. At young ($<15$) and very old ($>95$) ages, mortality rates from the experiment can be 0, which introduces infinite values into the log scale used in the figure, as well as infinite (x/0) or NaN (0/0). These values are not shown.

In the first experiment, we evaluated the effects of lineage survival and limited coverage of collateral kin by comparing genealogical subsets consisting only of direct ancestors across nine generations, representing incomplete surviving lineages, with whole simulated populations. Figure 1 compares the age-specific fertility and mortality rates for women in Sweden in 1900–05 (taken as an example from the middle of the period) and the evolution of the summary measures (TFR and $e_{0}$) for the different subsets over the whole period. Figure 6 in the Appendix compares the 1900–05 age-specific estimates with those from earlier (1800–05) and more recent (2000–05) years.

Regarding age-specific fertility rates, panel a of Fig. 1 suggests that the estimates from the genealogical subsets of direct ancestors (lines with squares and dots) are lower than those from the whole simulation (lines without shapes). Since these subsets only include the direct ancestors of the genealogists, the direct ancestors’ offspring (especially those who did not have children) are underrepresented in this type of genealogy. Since not all of the direct ancestors’ offspring are included in a given genealogist’s tree, their mothers may appear to have had fewer children than they gave birth to. For instance, if a woman had four children in the past, but only one of them is the direct ancestor of a genealogist, it would seem as though she had only one child. However, some of that direct ancestor’s siblings may also belong to another tree as direct ancestors of a different genealogist. Thus, this woman would be included in two or more family trees, and two or more of her children would be counted in the fertility estimates. Nevertheless, it is unlikely that all four of her children are direct ancestors of any of the genealogists, since the latter were a selected sample of individuals who survived to the present day.

Additionally, the estimates for all ages from the genealogical subset with duplicates (lines with squares) are lower than those from the subset without duplicates (lines with dots). This difference is more pronounced in the most distant periods (see Fig. 6 in Appendix). The number of possible duplicates is likely to increase the further back in time we go, since more distant ancestors are likely to be included in more family trees — and thus be common ancestors for more individuals — than more recent ancestors. For example, a female ancestor may appear in two or more genealogies as the mother of two or more different children (i.e. siblings), who are direct ancestors of different genealogists. If duplicates are removed, the mother would appear only once in the denominator, but both children would remain in the numerator, as they are not the same individual. Therefore, the age-specific fertility rates are higher in the subset without duplicates, as more duplicates are removed from the denominator than the numerator. In terms of timing, the age distribution is similar to that of the entire simulation, but fertility peaks at younger ages (25–30) in the genealogical subsets.

The extent of this bias over time can be examined by looking at the evolution of the fertility summary measure (i.e., TFR). Panel c of Fig. 1 shows the underestimation of fertility in genealogies comprising only direct ancestors (blue line with squares and olive line with dots) throughout the entire period. The TFR from the genealogical subset with duplicates is consistently close to one because every female direct ancestor has exactly one direct-ancestor child. Slight variations above or below one are probably due to approximations in the TFR calculation. This is calculated by dividing all births to women of a given age in a calendar year by the female population of that age at mid-year. Thus, the female population at risk is approximated by the female mid-year population.

However, the difference between the two subsets of direct ancestors and the whole simulation varies over time. The overall gap is larger before the twentieth century, when fertility was at its highest level, with a fluctuating total fertility rate (TFR) always above four children per woman. On average, including only direct ancestors leads to $-$2.4 and $-$1.5 children per woman (or -66% and -42%) in the genealogical subsets with and without duplicates, respectively, compared to the whole simulation. Before the fertility decline (approximated here as 1900), the effect was greater for the subset with duplicates, resulting in an average of 3.5 fewer children per woman (or a 76% decrease). During high fertility periods, the under-reporting of actual births in the genealogies of direct ancestors seems to be more pronounced, as the real number of births per woman was also higher. This results from the exclusion of collateral kin and, particularly ancestors without descent. Moreover, due to the research design, births to mothers belonging to extinct lineages were excluded from this and subsequent experiments.

As for age-specific mortality, the estimates derived from both genealogical subsets of direct ancestors, with and without duplicates, are relatively close to each other (see lines with squares and dots in panel b of Fig. 1. Therefore, including duplicates in the data does not seem to significantly affect the age distribution of deaths. Additionally, the estimates derived from the genealogies of direct ancestors closely resemble the estimates from the whole simulation after the age of 40. This pattern also holds for earlier and more recent years (see Fig. 6). This suggests that the shape of the mortality curve for adults and the elderly is not significantly affected by deriving the measures from direct surviving lineages. However, it is not possible to accurately estimate infant, child or young adult mortality from these subsets, since no deaths occurred before the age of 15, and deaths between the ages of 15 and 40 were underestimated. This is because, to be a direct ancestor of any genealogists, an individual must have survived to reproductive age, which necessarily excludes those who died before the age of 15. In the early twenty-first century, there were no deaths before the age of 35, probably because the parents of the genealogists did not experience any early deaths.

The exclusion of infant and child deaths, along with the underestimation of young adult mortality, can strongly bias the mortality summary measure, through the overestimation of life expectancy at birth (i.e., $e_0$) across the entire period (see panel d of Fig. 1). For females, this ranges from almost no bias (0.6 and 0.7 years) to 34 and 33 years higher for the subsets with and without duplicates, respectively. On average, this corresponds to an increase of $+33\%$ and $+32\%$ compared to the $e_0$ estimated from the whole simulation in the genealogical subsets with and without duplicates, respectively. The decreasing size of the bias may be related to the fact that the burden of infant and child mortality — which is not captured in genealogies of direct ancestors — had a greater positive impact on life expectancy estimates in past centuries than in more recent periods when improvements in mortality are mostly associated with old-age mortality.

### Experiment 2. Complete Surviving Lineages

**Fig. 2:**
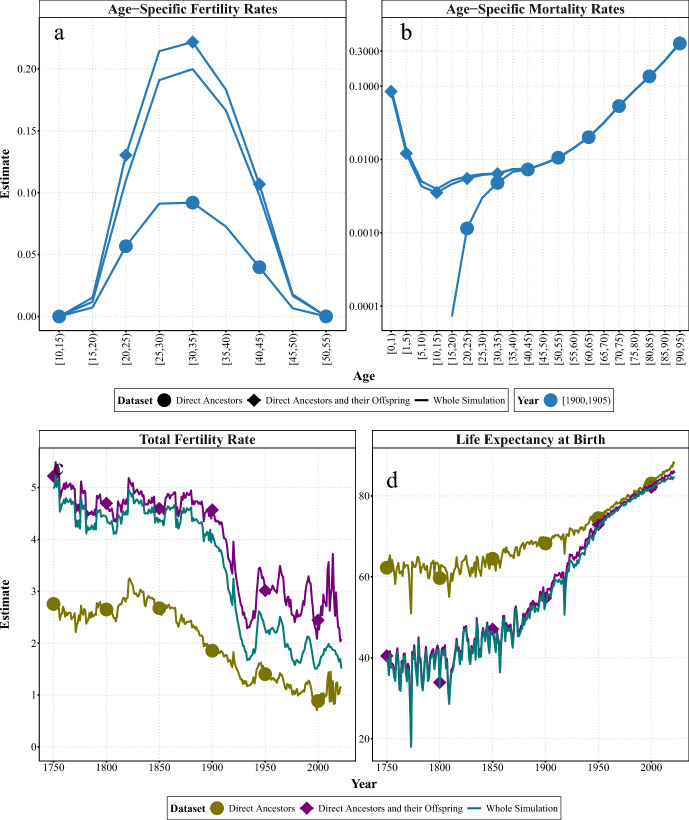
Experiment 2: Age-specific (1900–1905) and summary demographic measures (1751–2022) for women in Sweden, estimated from SOCSIM microsimulations and synthetic genealogical subsets comprising direct ancestors only and together with all direct ancestors’ offspring.* Notes* In each panel, the figure represents the means for each dataset. At young ($<15$) and very old ($>95$) ages, mortality rates from the experiment can be 0, which introduces infinite values into the log scale used in the figure, as well as infinite (x/0) or NaN (0/0). These values are not shown.

In the second experiment, we examined the effect of lineage survival by comparing complete and incomplete surviving lineages with the whole simulated population. Complete surviving lineages included the direct ancestors from Experiment 1 and all their offspring. From this experiment onwards, we removed all duplicates from all calculations. While the lineages include some relatives from the same generation as the genealogist (their siblings), the majority belong to previous generations. Thus, demographic events corresponding to more recent years are only partially covered, and the results for these years should be treated with caution. For the sake of readability, we compare the estimates from genealogical subsets comprising only direct ancestors (incomplete surviving lineages) and direct ancestors with all their offspring (complete surviving lineages) with those from the whole simulated population. For women in Sweden, Fig. 2 compares the age-specific fertility and mortality rates for women in Sweden in 1900–05, and the evolution of the summary measures (TFR and $e_{0}$) over the whole period. Figures 7 and 8 in the Appendix compare the estimates based on the subsets that gradually add one generation of the direct ancestors’ offspring.

Concerning age-specific fertility, panel a of Fig. 2 shows that the estimates from the genealogical subsets with direct ancestors and their offspring (diamond-marked lines) are larger than the estimates from the whole simulation (unmarked lines) and the genealogical subset of only direct ancestors (dot-marked lines). As we go back in time, the overestimation of fertility is due to the inclusion of the births of the offspring of more distant generations of direct ancestors (see Fig. 7 in Appendix.). This trend can also be seen over time; see panel c of Fig. 2. Estimates of TFR based on genealogies of direct ancestors and their offspring (purple line with diamonds) are larger than the estimates from the whole simulation and the genealogies of only direct ancestors, implying an overestimation by $+0.3$ children per woman (or a $+6\%$ increase of TFR) before the fertility decline and $+0.5$ children per woman (or a $+21\%$ increase) over the whole period. Here, the bias is larger over the most recent periods. Figure 8 in Appendix illustrates the differences in the accuracy of the estimates as an additional generation of ancestors’ offspring is included.

Regarding age-specific mortality, the estimates for adult and old-age mortality from the genealogical subsets comprising either direct ancestors or direct ancestors and their offspring (lines marked with dots and diamonds, respectively) are quite similar (see panel b of Fig. 2). This also holds for earlier and more recent periods (see Fig. 7). However, the estimates from the genealogical subset including the direct ancestors’ offspring are now much closer to the estimates from the whole simulation in terms of level and timing, even for infant and young-age mortality, which does not exist in the subset of direct ancestors only. Although the estimates are slightly lower for younger age groups, including all the offspring of direct ancestors enables us to account for individuals who died young and did not have children, but who still belong to a given surviving lineage. This improves the estimation of infant, child, or young adult mortality compared to genealogies of direct ancestors.

The inclusion of direct ancestors’ offspring also affects the mortality summary measure ($e_{0}$). As shown in panel d of Fig. 2, estimates of life expectancy at birth from the genealogical subset with direct ancestors’ offspring are very close to those derived from the whole simulation over the entire period. However, the former are still slightly higher, resulting in an overall overestimation of 0.9 years (or $+1.8\%$). Thus, including all direct ancestors’ offspring significantly improves the accuracy of demographic estimates based on genealogies, as each generation provides more information about the demographic events of each period. Figure 8 in the Appendix shows the changes in the estimates with the inclusion of an additional generation.

### Experiment 3. Surviving Lineages with Selective Omission

**Fig. 3:**
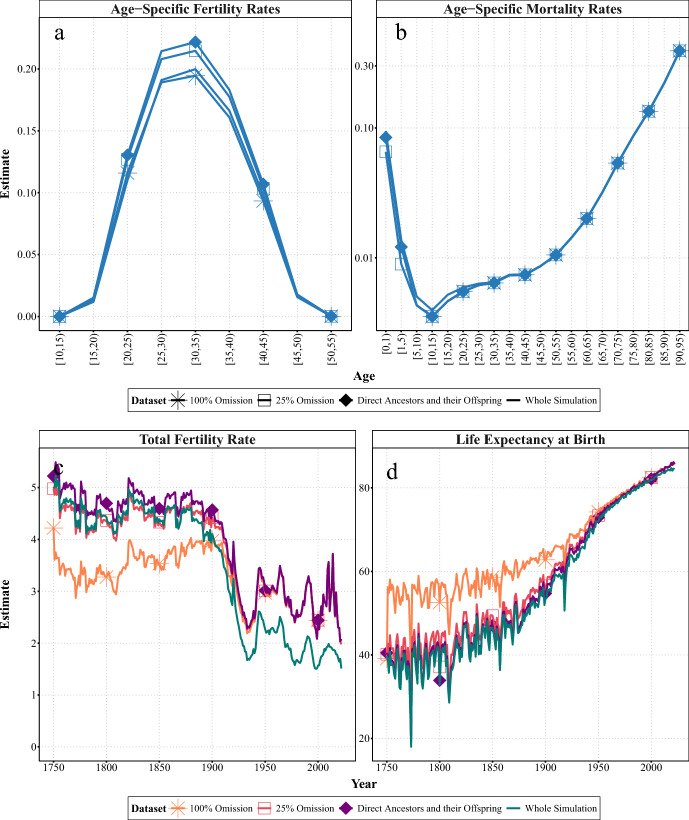
Experiment 3A: Age-specific (1900—1905) and summary demographic measures (1751—2022) for women in Sweden, estimated from SOCSIM microsimulations and synthetic genealogical subsets, which included all direct ancestors and their offspring, or omitted some proportions of children who died before the age of five.* Notes* In each panel, the figure represents the means for each dataset. At young ($<10$) and very old ($>95$) ages, mortality rates from the experiment can be 0, which introduces infinite values into the log scale used in the figure, as well as infinite (x/0) or NaN (0/0). For the dataset with $100\%$ omission all mortality rates below age 10 are 0. These values are not shown.

**Fig. 4:**
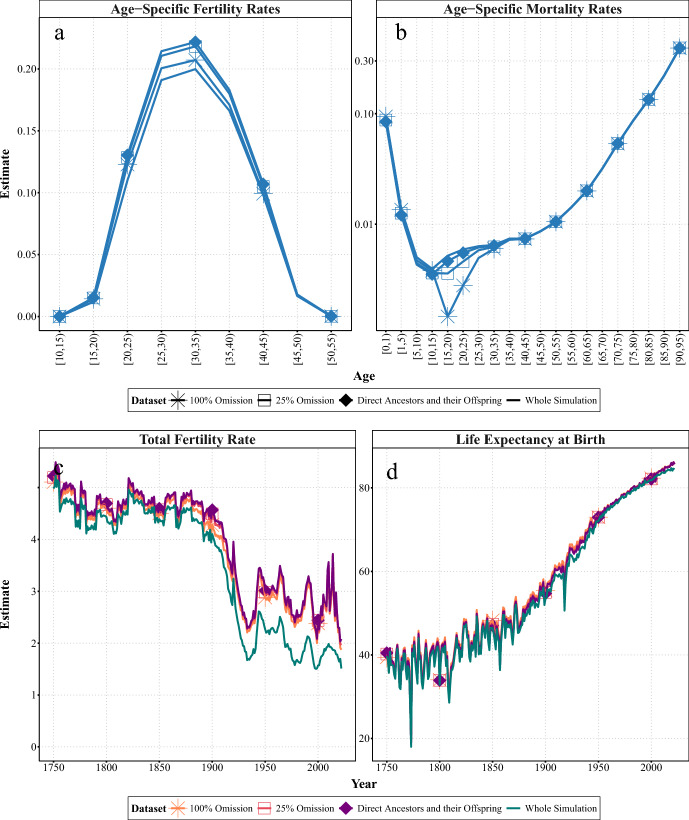
Experiment 3B: Age-specific (1900—1905) and summary demographic measures (1751—2022) for women in Sweden, estimated from SOCSIM microsimulations and synthetic genealogical subsets, which included all direct ancestors and their offspring, or omitted some proportions of childless women.* Notes* In each panel, the figure represents the means for each dataset. At very old ($>95$) ages, mortality rates from the experiment can be 0, which introduces infinite values into the log scale used in the figure, as well as infinite (x/0) or NaN (0/0). These values are not shown.

**Fig. 5:**
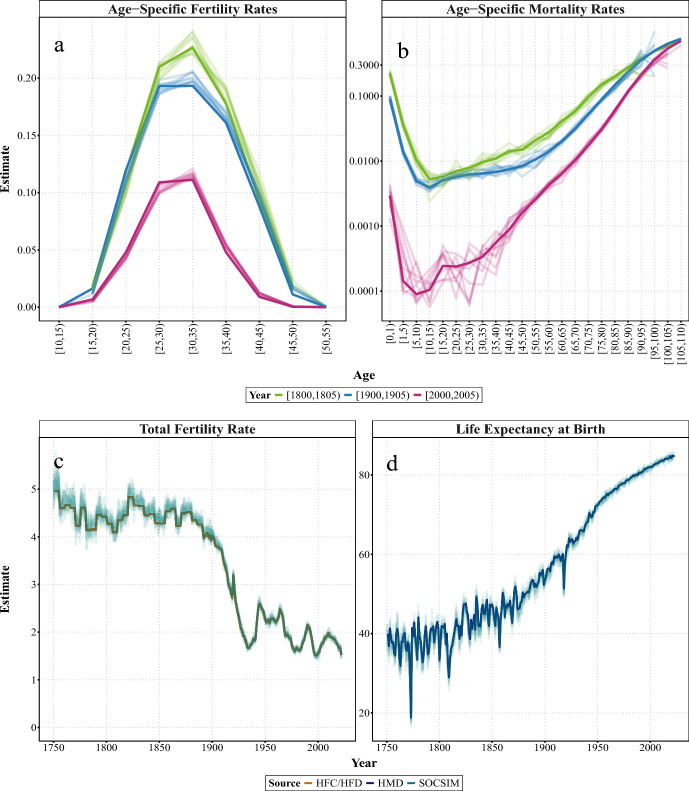
Age-specific and summary demographic measures for women in Sweden, retrieved from the Human Fertility Collection (HFC), the Human Fertility Database (HFD), the Human Mortality Database (HMD), and SOCSIM outputs.* Notes* The bold lines correspond to HFC/HFD and HMD estimates and the transparent lines to the 10 SOCSIM simulations. At young and very old ages, mortality rates from the simulations can be 0, which introduces infinite values into the log scale used in the figure, infinite (x/0) or NaN (0/0). These values are not shown.

**Fig. 6:**
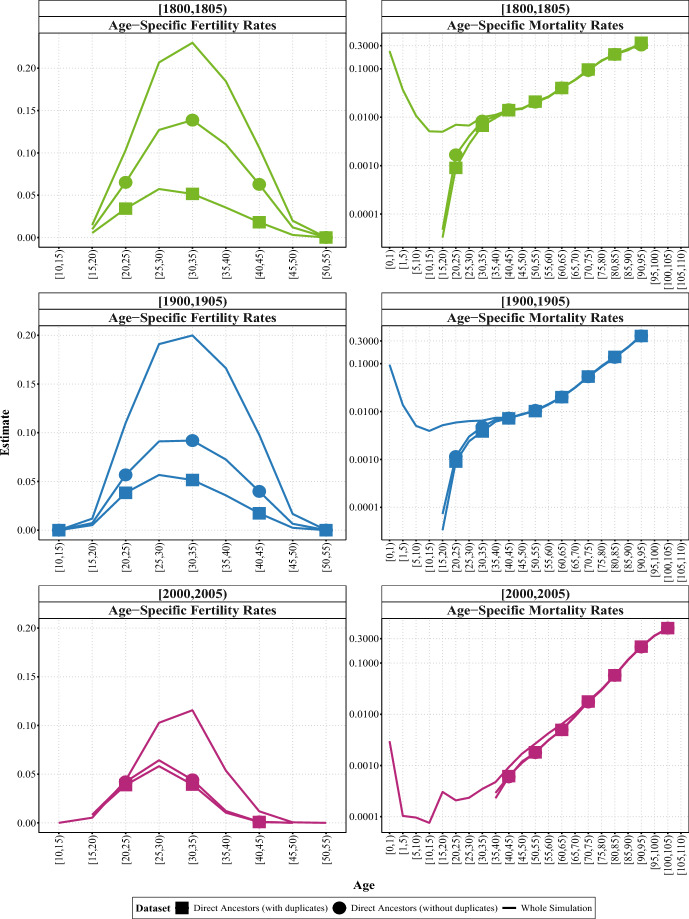
Experiment 1: Age-specific demographic measures for women in Sweden, estimated from SOCSIM microsimulations and synthetic genealogical subsets comprising only direct ancestors (with and without duplicates) in three selected periods.* Notes* In each panel, the figure represents the means for each dataset. At young and very old ages, mortality rates from the simulations can be 0, which introduces infinite values into the log scale used in the figure, infinite (x/0) or NaN (0/0). These values are not shown.

**Fig. 7:**
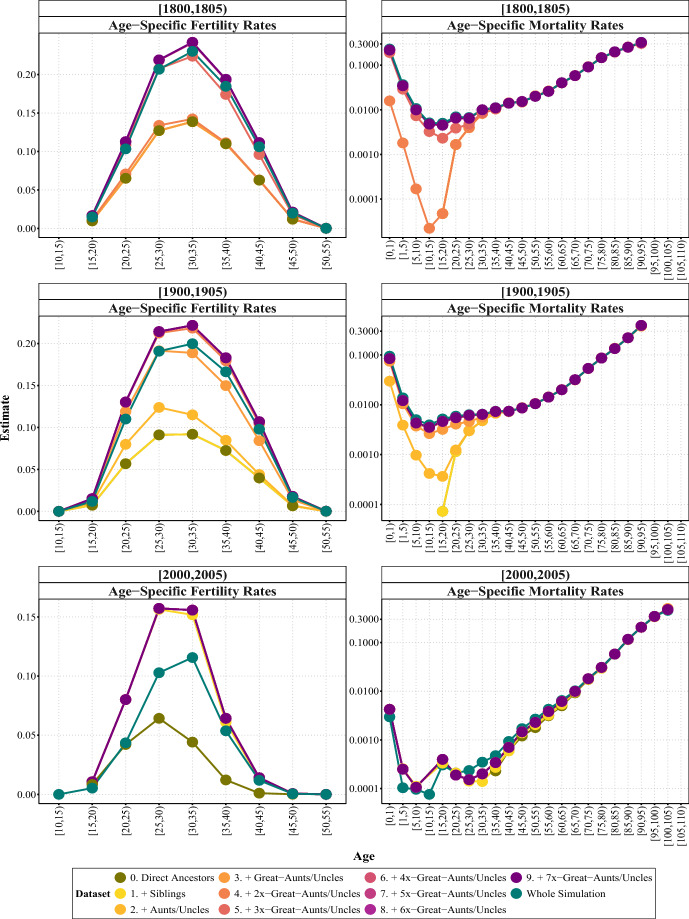
Experiment 2: Age-specific demographic measures for women in Sweden, estimated from SOCSIM microsimulations and synthetic genealogical subsets comprising only direct ancestors and with progressive inclusion of direct ancestors’ offspring in three selected periods. *Notes* In each panel, the figure represents the means for each dataset. At young and very old ages, mortality rates from the simulations can be 0, which introduces infinite values into the log scale used in the figure, infinite (x/0) or NaN (0/0). For the dataset with $100\%$ omission of early-deceased children all rates below age 10 are 0. These values are not shown.

**Fig. 8:**
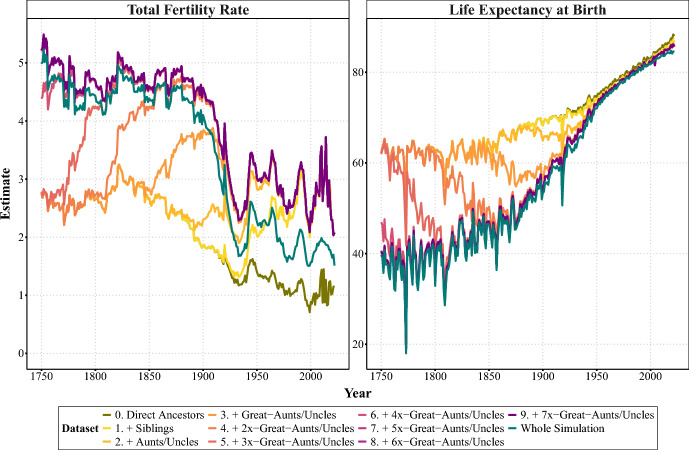
Experiment 2: Summary demographic measures for women in Sweden estimated from SOCSIM microsimulations and synthetic genealogical subsets comprising only direct ancestors and with progressive inclusion of direct ancestors’ offspring.* Notes* In each panel, the figure represents the means for each dataset.

**Fig. 9:**
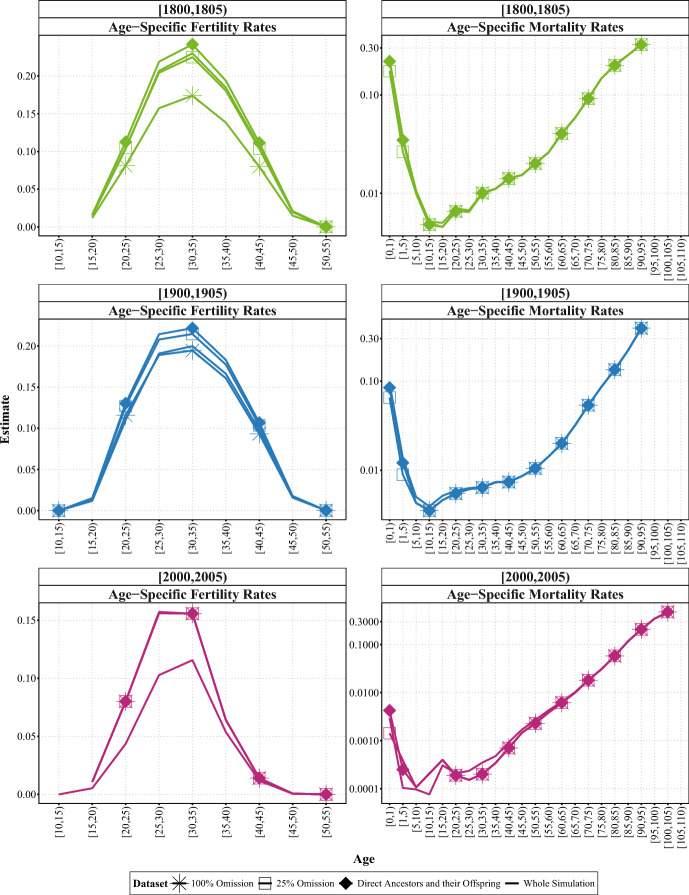
Experiment 3A: Age-specific demographic measures for women in Sweden, estimated from SOCSIM microsimulations and synthetic genealogical subsets, which included all direct ancestors and their offspring, or omitted some proportions of children who died before the age of five in three selected periods.* Notes* In each panel, the figure represents the means for each dataset. At young and very old ages, mortality rates from the experiment can be 0, which introduces infinite values into the log scale used in the figure, as well as infinite (x/0) or NaN (0/0). For the dataset with $100\%$ omission all mortality rates below age 10 are 0. These values are not shown.

**Fig. 10:**
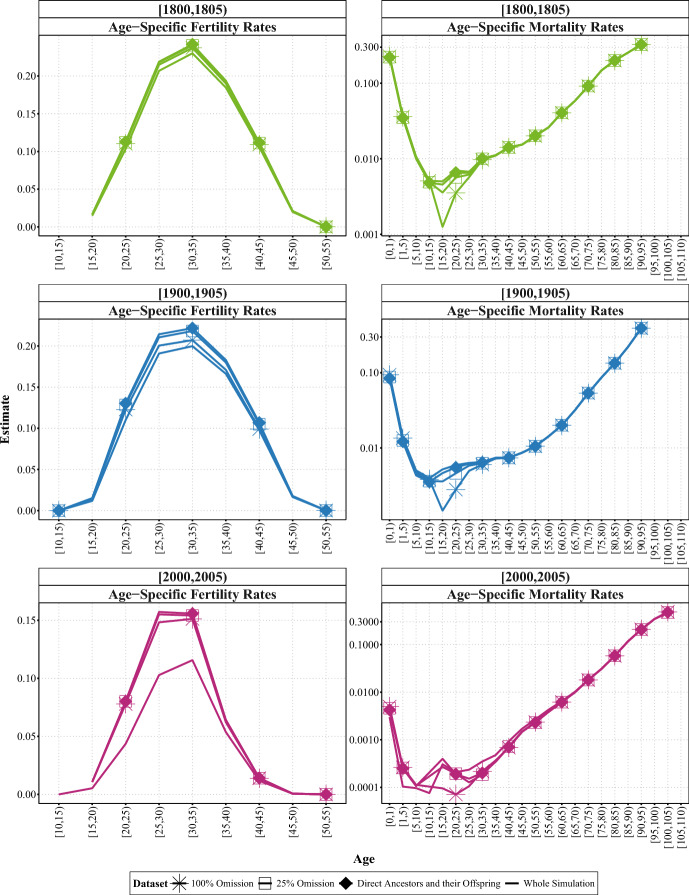
Experiment 3B: Age-specific demographic measures for women in Sweden, estimated from SOCSIM microsimulations and synthetic genealogical subsets, which included all direct ancestors and their offspring, or omitted some proportions of childless women in three selected periods.* Notes* In each panel, the figure represents the means for each dataset. At very old ($>90$) ages, mortality rates from the experiment can be 0, which introduces infinite values into the log scale used in the figure, as well as infinite (x/0) or NaN (0/0). These values are not shown.

In our third experiment, we examined the effects of selective omission in complete surviving lineages by simulating the exclusion of children who died young and childless women due to missing records or memory-related issues. Using a similar approach for children and women separately, we analysed the effects of omitting these individuals from the genealogies of direct ancestors and their offspring. Figures 3 and 4 show age-specific fertility and mortality rates for women in Sweden in the period 1900–1905, and the evolution of the summary measures (TFR and $e_{0}$) over the whole period, derived after omitting different proportions of children who died before the age of five or childless women, respectively. For clarity, only the estimates with $25\%$ and $100\%$ omission are shown. We compared these estimates with those derived from the complete surviving lineages and the whole simulation. In both cases, removing these individuals biases the estimates in the same direction, but the magnitude is larger when omitting children who died early. We also explored using an age threshold of one for the early-deceased children, but the results (not included here) showed patterns very similar to those obtained using an age threshold of five, except for the age-specific mortality rates below ages one and five. Figures comparing the age-specific estimates from 1900–05 with those from earlier (1800–05) and more recent (2000–05) years are provided in Figs. 9 and 10 in Appendix.

Figure 3 compares the estimates derived after omitting some children who died before the age of five from the genealogies of direct ancestors and their offspring. Regarding fertility, panel a of Fig. 3 shows that the age-specific rates from the genealogical subsets with omitted early-deceased children are lower than those from the complete subsets of direct ancestors and their offspring (lines with diamonds). This bias increases as the percentage of omission increases. The age distribution is almost unaffected. This also applies to earlier periods when fertility and mortality rates were high. However, in recent years, when infant and child mortality rates have been very low, the difference is almost imperceptible (see Fig. 9).

Looking at the changes in the summary measure over time, panel c of Fig. 3 suggests that the effect of omitting early deceased children on underestimating fertility increases the further back in time one goes. This bias increases in proportion to the percentage omitted, particularly before the twentieth century, when fertility and under-five mortality were both high in Sweden. Before 1900, an omission of $25\%$ of children who died in early childhood on average leads to an underestimation of $-0.04$ children per woman (or a reduction of $-0.73\%$ in the TFR) compared to the whole simulation, while $100\%$ of omission on average leads to an underestimation of $-0.94$ children per woman (or a reduction of $-21\%$ in the TFR). Under-five mortality was particularly high in earlier centuries and began to decline in Sweden from the eighteenth century onwards. Therefore, the further back in time we go, the larger the number of children who were ever born and who would be missing from the genealogies if those who died at an early age were omitted. From the twentieth century onwards, the gap to the complete genealogies decreases, becoming minimal in recent decades.

For mortality, the age-specific estimates are only lower for ages zero to one and one to five, with $100\%$ omission resulting in non-existent rates (see panel b of Fig. 3). This pattern is evident in both earlier and more recent years (see Fig. 9 in Appendix). However, omitting children who died early leads to a significant overestimation of life expectancy at birth ($e_{0}$) (see panel d of Fig. 3). This effect increases significantly the further back in time we go and the greater the proportion of omission. Omitting $25\%$ of children who died early can lead to an overestimation of $e_{0}$ by up to 2.6 years (i.e., $+5.8\%$), while omitting $100\%$ can lead to an overestimation of $e_{0}$ by up to 9.3 years (i.e., $+22\%$).

Figure 4 compares the estimates obtained after omitting childless women from the complete surviving lineages. For fertility, this omission resulted in a slight underestimation of the age-specific rates compared to genealogies of direct ancestors and their offspring (see panel a of Fig. 4). The same is true for earlier and more recent years (see Fig. 10). Again, the age distribution is unaffected. This trend can be observed when examining the evolution of the TFR. Omitting childless women results in slightly lower estimates of TFR compared to those derived from the complete genealogies (see panel c of Fig. 4). Otherwise, there is no significant change in the magnitude of the bias over time.

As for mortality, the age-specific estimates are lower than those from the complete surviving lineages and the whole simulation, but only for ages 15—35 (see panel b of Fig. 4). This is true not only for 1900—05, but also for previous and more recent centuries (see Fig. 10 in Appendix). However, omitting childless women has a relatively small impact on the overestimation of life expectancy at birth ($e_{0}$), which increases slightly as larger proportions of childless women are omitted (see panel d of Fig. 4). Over the whole period, omitting $25\%$ of childless women leads to an overestimation of $e_{0}$ by up to 1.05 years (i.e., $+2.1\%$), while omitting $100\%$ leads to an overestimation of $e_{0}$ by up to 1.5 years (i.e., $+9.57\%$).

## Discussion

Genealogies are promising sources for research on historical and kinship demography. However, these data have not yet been leveraged to their full potential, as the biases affecting their representativeness have not been fully understood. In this study, we conducted a series of experiments on synthetic populations to assess how three main sources of bias in ascendant genealogies can affect the accuracy of demographic estimates. Using the SOCSIM demographic microsimulation programme, and taking Sweden (1751–2022) as a case study, we generated fully recorded synthetic populations that were used as benchmarks for our experiments on ascendant genealogies. From these simulated populations, we traced the family trees of a group of hypothetical genealogists alive in 2022, and extracted different genealogical subsets that reproduced the sources of bias. We explored selection in genealogies reconstructed from lineages that survived to the present, which resulted in the exclusion of extinct lineages. Based on these surviving lineages, we also considered two further sources of bias: the limited coverage of collateral kin, which restricts the analysis to direct ties, and selective omission, whereby certain individuals, such as children who died young and women who never had children, are excluded from the lineages.

Our analysis highlights three key points. Firstly, the accuracy of demographic estimates based on ascendant genealogies is affected by the exclusion of extinct lineages and the completeness of family trees. Here, this is approximated by comparing the inclusion of direct ancestors alone with the addition of collateral kin. Including the offspring of direct ancestors to create complete surviving lineages tends to overestimate fertility while reducing the bias in mortality estimates, bringing them closer to the true values. Secondly, the magnitude of these effects appears to vary over time, particularly between periods characterised by high and low fertility and mortality rates. This suggests that the effects are not linear. Thirdly, the omission of certain individuals who are often under-recorded in genealogies, such as early-deceased children and childless women, seems to follow a similar pattern of variation, especially pronounced for children.

According to previous research, genealogical data tend to show higher fertility and lower mortality. Our results suggest that this largely depends on the completeness of family trees. Including only direct ancestors can lead to an underestimation of the number of births a woman might have had by $-42\%$ and an overestimation of life expectancy at birth by $+33\%$, mainly due to missing infant, child and young adult mortality. However, middle-age and old-age mortality are well captured even in these incomplete genealogies. In Experiment 2, after the offspring of all direct ancestors were included, the fertility rate derived from genealogies was found to be $+21\%$ higher than that derived from the whole simulation, while the overestimation of life expectancy at birth was reduced to $+1.8\%$ over the entire period. These changes in the estimates suggest that extending the kinship network in the family tree is essential for producing accurate demographic estimates based on genealogies.

This study has some limitations that we would like to acknowledge. Firstly, our analysis is based on synthetic populations, which differ from real populations and cannot fully capture their complex dynamics and structures. Familial transmission of fertility and mortality behaviour is not considered because the input data are only available by sex and age.^1^ Thus, beyond the individual stochasticity resulting from the microsimulation, there is no predefined clustering of families with better or worse demographic conditions. Similarly, the microsimulation model has no option to account for the relationship between fertility and mortality.

Although ignoring the correlations between demographic behaviour across families, and between fertility and mortality, may distort estimates of the bias, this is a known limitation of most microsimulation studies. The absence of familial clustering could result in a more homogeneous distribution of kin, leading to an underestimation of the proportion of individuals with either no relatives or many (Ruggles, 1993). This could produce fewer extreme cases, with some lineages that would otherwise become extinct surviving in the simulation. However, not accounting for the correlation between fertility and mortality could obscure differences in lineage survival. Previous research has examined the potential trade-off between fertility and lifespan, yielding mixed results in historical populations and more consistent findings in contemporary ones. While historical evidence suggests negative, positive or neutral associations depending on the context, recent studies more often report higher mortality among childless and high-parity women (Kuningas et al., 2011). Assuming that childless women have shorter lifespans in certain contexts, their exclusion from genealogies could lead to an underestimation of observed mortality rates. Similarly, if high-parity women are more likely to experience shorter lifespans, potentially due to maternal mortality or health deterioration following multiple pregnancies, their greater representation in surviving lineages could also affect observed mortality patterns. As our simulation does not model such correlations, these mechanisms are not reflected in the results. However, the opposing effects that these factors may have on lineage survival could offset each other when average outcomes are examined. Therefore, any resulting bias is expected to be limited.

Secondly, due to the lack of reliable age-specific marriage rates by sex for the entire study period, we modelled lifelong partnerships and fertility behaviour using the ‘marriage after childbirth’ option in ‘rsocsim’. While this approach may oversimplify real-world dynamics and the increasing complexity of family structures, it allowed us to address the absence of accurate historical data on marriage. This approach performs reasonably well in periods when marital fertility was the norm, producing relatively accurate estimates of female fertility. However, it may introduce biases in contexts where childbearing outside marriage is prevalent, or where family structures involve multiple partners, step-families or complex dynamics. Yet these issues are not the focus of this analysis.

Thirdly, age-specific fertility rates by birth order, which would allow for a more accurate parity distribution from the simulation, are typically unavailable over the long term. Therefore, while synthetic parity distributions are overall reasonable, they may not perfectly mirror empirical ones, which could affect the estimates of the biases. In the absence of parity-specific data, individuals with zero or only one child are likely to be overrepresented, while high-order births are likely to be underrepresented (Calderón-Bernal et al., 2025). The simulation’s overrepresentation of null and low parities may slightly amplify the bias associated with lineage survival and, more strongly, the selective omission of childless women. Conversely, the underrepresentation of high parities may reduce the effect of limited coverage of collateral kin. Otherwise, the simulation’s parity distribution is primarily determined by the input fertility rates, with no incorporation of parity-specific assumptions. It is therefore reasonable to hypothesise that a similar distortion, overestimating lower parities and underestimating higher parities, would occur in other populations, and across periods, simulated with different data with no parity-specific information. In such cases, the magnitude of the distortion in the estimates of biases may vary with the population’s fertility, whereas its direction would likely remain similar.

Fourthly, our research design involves randomly selecting genealogists from among individuals who were alive at the end of the simulation. Therefore, based on our synthetic populations, we can only reproduce the selection resulting from the survival of certain lineages until the time of genealogical reconstruction, rather than that resulting from other factors such as better demographic or socioeconomic conditions. Consequently, our analysis focuses solely on structural biases in genealogical data, meaning that other biases identified in the literature, such as the accuracy (Minardi et al., 2024) or elite bias (Stelter & Alburez-Gutierrez, 2022), are not considered.

Fifthly, our experiments are based on the ideal scenario of creating extensive, fully documented family trees in which genealogists can trace the demographic information of all ancestors and their offspring nine generations backwards without restriction. In reality, however, it can be challenging for genealogists to obtain comprehensive and reliable information on distant ancestors, as data from earlier periods is often imprecise, incomplete or unavailable, and tends to be more accessible for larger or wealthier families. Apart from testing the omission of deceased children and childless women from surviving lineages (Experiment 3), we do not assess the bias resulting from imprecise or incomplete data in genealogies (so-called precision bias). This may also limit the scope of our results.

Finally, the definition and implementation of ascendant genealogy in this research may affect the results, as including certain types of kin may result in incomplete demographic information from recent periods. This is particularly likely when descendants, such as the children, nieces, nephews, or grandchildren of the genealogists, could have provided information. Affinal and in-law relatives were excluded to restrict the genealogical reconstruction to consanguinity.

Our study of fully recorded synthetic genealogies offers valuable insights to researchers using genealogical data for historical demographic research. We showed that using direct surviving lineages alone to derive demographic estimates can produce inaccurate results for measures such as life expectancy at birth, which are sensitive to mortality at early and young ages. However, mortality at older ages is still well represented. Including the offspring of direct ancestors in ancestors-only genealogies improves the accuracy of estimates, particularly for mortality, by better capturing deaths at younger ages. This highlights the importance of the completeness of family trees within ascendant genealogies, which should be carefully assessed before using these data. Researchers working with these datasets should always compare estimates derived from genealogies with those obtained from other traditional sources, to understand the magnitude and direction of biases in the data. As suggested in this study, underestimation of fertility may indicate the exclusion of certain types of ancestors’ offspring from genealogies, particularly during periods of high fertility. Significant overestimation of life expectancy at birth in past centuries likely suggests underestimation of infant and child mortality, although estimates can become more accurate when conditioning on survival to age five, ten, and beyond. Finally, ascendant genealogies can hardly account for contemporary demographic events. For this reason, researchers should carefully consider the period for which sufficient high-quality data exists and for which the kinship networks are complete enough to ensure that the vital events of their members are representative.

Future research could expand the analytical scope of this study by applying our simulation framework to a broader range of demographic measures. For instance, studies could examine parity distributions or mortality rates at different ages, or adopt a cohort perspective. This could enhance our understanding of the extent to which diverse measures are more or less sensitive to the types of bias commonly present in genealogical data. Additionally, the impact of family clustering and correlations on demographic summary measures in both genealogies and population-level data can be examined.

Moreover, our simulation-based approach paves the way for empirical validation by facilitating comparisons with genealogical and official data. Although our study was not designed to validate genealogical sources directly, it does simulate the decision-making processes commonly employed by genealogists when reconstructing family trees. These include focusing on direct lineages, deciding whether to include or exclude collateral kin, and omitting individuals who died young or had no descendants. Future research could investigate how often such practices occur in actual datasets, and the extent to which they contribute to observable biases in demographic measures. For instance, it could examine whether genealogists systematically prioritise certain types of kin and whether omitting individuals (e.g. those who died as infants or were childless) reveals biases that are consistent or vary across countries, historical periods, or datasets. Additionally, future work could examine the significance of ancestral duplication in real-world genealogies. This would include both pedigree collapse, where the same ancestor appears multiple times within a single lineage, and duplication across genealogies, where the same individual appears in different family trees. While such duplicates are to be expected in any lineage reconstruction, their impact is likely to vary depending on the population size and the depth of genealogical reconstruction. Investigating how often these forms of duplication occur in crowdsourced datasets and how they affect demographic estimates would clarify the empirical implications of our findings.

Furthermore, comparing demographic rates derived from online genealogical data with those produced by our experiments could help isolate the impact of structural biases in ascendant genealogies from other sources of biases. Such comparisons would clarify whether discrepancies in empirical genealogies primarily result from the decisions of genealogists, their data curation strategies, or the limitations of historical records. It would also allow researchers to evaluate the extent to which biases observed in simulations correspond with those documented in comparisons between genealogical and official datasets. This would help to bridge the gap between data quality assessments based on simulations and those based on empirical data.

## Supplementary Information

Below is the link to the electronic supplementary material.

Supplementary file 1 (csv 4437 KB)

## Data Availability

The code to retrieve the data, run the microsimulations and reproduce the results is available online: https://github.com/liliana-calderon/SOCSIM_Genealogies

## Appendix

See Figures 5, 6, 7, 8, 9and 10

## Footnotes

1 In the current version of rsocsim, a heterogeneous fertility option can be enabled to allow for heritability through the maternal line. However, the default option significantly underestimates fertility, particularly during periods when it was high, requiring substantial calibration of the rates.
